# Evidence-Based Practice for the Use of pH Indicator Paper Strip in Oral Medicine: A Literature Review

**DOI:** 10.7759/cureus.62797

**Published:** 2024-06-20

**Authors:** Baraa Shamsi-Basha, Raphaelle Bernard-Garbati, Emmanuel Mortier, Anne Grasland, Karim Lachgar, Alp Alantar

**Affiliations:** 1 Oral Surgery, Max Fourestier Hospital, Nanterre, FRA; 2 Internal Medicine, Max Fourestier Hospital, Nanterre, FRA; 3 Rheumatology, Max Fourestier Hospital, Nanterre, FRA; 4 Diabetology, Max Fourestier Hospital, Nanterre, FRA

**Keywords:** early chilhood caries, periodontitis, treatment, pathology, disease, ph indicator paper, ph indicator strips, oral cavity ph, salivary ph

## Abstract

The objective of our article is to review the literature and collect the advice of specialists for the evaluation of the reliabilityand appropriate indications of the use of pH paper in oral medicine practice. The literature pertaining to the use of pH paper in oral medicine practice was reviewed, and appropriate indications were suggested by a French multidisciplinary working group of specialists and validated by a lecture committee. By screening PubMed/MEDLINE from 1911 to March 2024, we found 621 articles. All abstracts were read, 22 articles were selected for full-text reading, and 12 were ultimately included in the review. Three more articles from other sources were included. Thus, 15 articles constituted the literature review; seven papers from PubMed/MEDLINE focusing on how to restore the salivary pH balance in cases of periodontitis and early childhood caries (ECC) completed the review. It is concluded that the lack of sensitivity of pH paper must be underlined. A low pH is a cofactor leading to oral pathologies, and the use of pH paper constitutes an easy diagnostic instrument in patients with pH variations correlated to leukemia, diabetic mellitus, or orofacial radiotherapy. The evaluation of salivary pH using pH paper may be used as a quick chairside test, specifically in cases of ECC and uncontrolled severe periodontitis. Early diagnosis of salivary low pH range in children as well as periodontitis with deep pocket associated with a low pH range in adults should lead to the supply of fluoride and prescript sodium bicarbonate-containing dentifrices, respectively. In children, the use of a chewable toothbrush may help reduce plaque and elevate salivary pH.

## Introduction and background

The pH scale is logarithmic and inversely indicates the activity of hydrogen ions in a solution. The salivary pH may be correlated to the oral and general health status, such as neurological or oral motor dysfunction [1,2]. Salivary components may suffer variations that can be detected by chemical determinations. Low pH levels have been correlated with dental caries prevalence in young diabetics, according to López et al. [2,3]. In periodontitis, Galgut [4] established a highly significant correlation between the periodontal pocket and pH range and a significant correlation between the deep pocket and low pH range.

The use of pH paper seems to have been partially abandoned in oral medicine clinical practice. Nowadays, the use of electrodes in oral investigations has been strongly developed. A measurement by paper strip has been developed as an assessment tool that is noninvasive, painless, easily collected, and, in general, less expensive than other diagnostic tools. These small strips of paper are coated with a special dye that changes color when it comes into contact with acidic or basic substances. The aim of this review is to evaluate the reliability and indications of the use of pH indicator paper strips in oral health and medical status.

## Review

Preliminary search

The literature review of the indications of pH paper use was performed by searching the PubMed/MEDLINE database. The screening included articles published between 1911 and March 2024 in French or English using the following keywords: ((Salivary pH) or (pH saliva) or (oral cavity pH)) and ((pH indicator strips) or (pH indicator paper)) and ((disease) or (pathology) or (treatment)).

Eligibility criteria

The case study, case report, and case series articles were selected for the review if they respected the following inclusion criteria: (a) measured the salivary pH; (b) used a pH indicator paper; (c) on patients with oral or systemic diseases or under pharmacological treatment. These criteria were applied with no restriction on the level of scientific evidence. Abstracts and full-text articles relating to the measurement of salivary pH with other techniques than pH paper and/or in patients without pathology or treatment were excluded. Studies in other languages than English or French were excluded. Further research using the references quoted in the full-text articles was carried out. A second screening of articles published between 1983 and 2022 focusing on how to restore the salivary pH balance - matching the following keywords: (periodontitis), (early childhood caries), and (sodium bicarbonate) - completed the literature review.

Data processing

The articles and guidelines were analyzed, and a first set of data was proposed for peer review by a working group (see Appendix A for members of the working group). The revised recommendations were then submitted to a multidisciplinary lecture committee (see Appendix A for members of the lecture committee). The final document was modified according to the multiple critical appraisals of the two groups. The literature review was followed by critical appraisal by a multidisciplinary working group in order to classify data according to the levels of scientific rigor (levels 1, 2, 3, and 4) [5].

Results 

**Figure 1:**
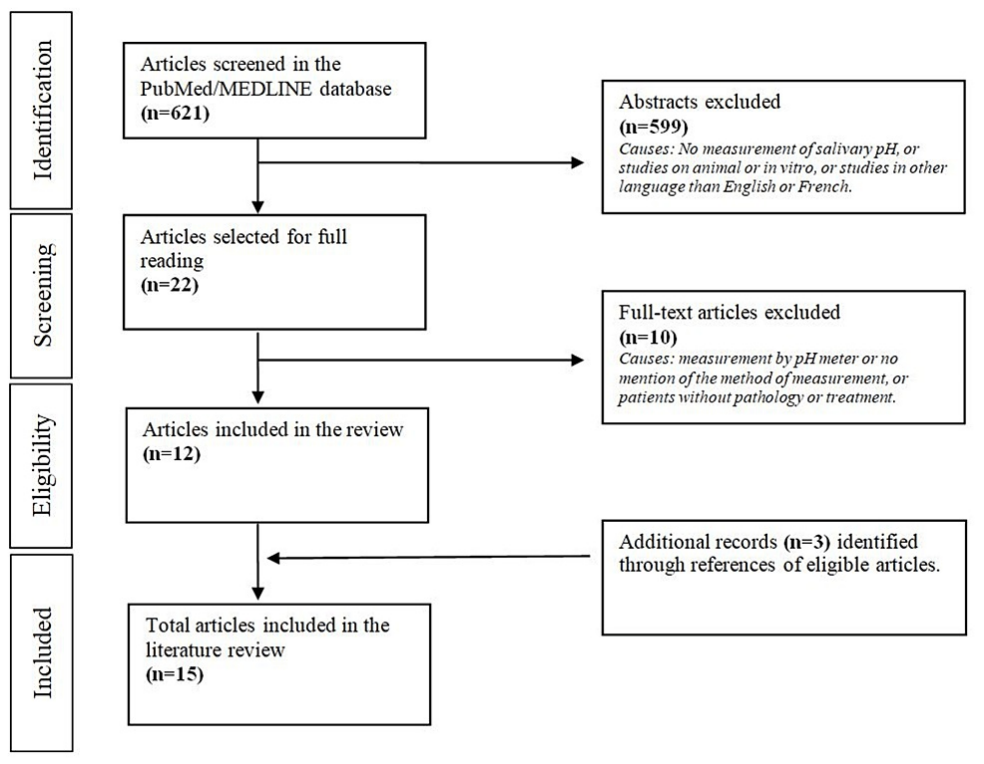
Study selection methodology

The study selection methodology is presented in Figure 1.

The PubMed/MEDLINE screening was performed until March 2024, and we found 621 articles. All abstracts were read; 22 articles were selected for full reading, and 12 were included in the review. Additional papers (n = 3) identified from references in the full-text articles were selected. A total of 15 articles were included in the literature review [1,4,6-18]. In addition, seven papers focusing on how to restore the salivary pH balance completed the review [3,19-24].

Method of Measurement

Of the 15 publications reviewed, 10 measured the salivary pH by first collecting the saliva in a container with a spitting or drooling method. Most of these studies (eight out of 10) collected unstimulated saliva (Table 1). In some studies, the collection of unstimulated saliva was done after at least one hour of fasting, with a water rinse before collection. Stimulated saliva was obtained after asking the patient to chew on a piece of parafilm for five minutes.

**Table 1:** Overview of selected publications * Hierarchy of levels of scientific rigor [5]: level 1 (high level): RCTs with high statistical power, meta-analyses of RCTs, and decision analysis based on studies with high methodologic quality; level 2 (intermediate level): RCTs with low statistical power, nonrandomized comparative studies with high-quality methodology, and cohort studies; level 3 (weak level): case-control studies; level 4 (weak level): comparative studies with important bias, retrospective studies, sample of cases, and descriptive epidemiologic studies (cross-sectional or longitudinal). GERD, gastroesophageal reflux disease; RCT, randomized controlled trial

Publication	Population of patients	Paper’s method of use	Type of pH paper	Level of evidence^*^
Laudenbach et al. (1974) [1], Case series	Patients (n = 167) with various systemic or oral pathologies	pH paper on the top of the tongue, ostium of Wharton, and Stenon ducts (anatomical landmark)	Unspecified	Level 4
Galgut (2001) [4], Cross-sectional study	Patients (n = 42) with gingivitis or periodontitis	pH paper cut into small pieces, placed interdentally in one healthy site, in one inflammatory site without pocketing, in one site with pocketing, on the floor of the mouth, and in the midline of the soft palate for 30 seconds	Two types of pH paper at each site: one with a pH range of 1-5 and one with a pH range of 5-9	Level 4
Ramya et al. (2015) [6], Case-control study	Patients (n = 90) with cancer prior to treatment vs. patients with cancer undergoing treatment vs. healthy controls	Two to three drops of unstimulated whole saliva on the strip; saliva collected by spitting five minutes after the water rinse and after at least an hour without drinking and eating	Merck Serono indicator strips, USA	Level 3
Kapoor et al. (2019) [7], Cross-sectional study	Three- to 14-year-old children (n = 220) with acute lymphoblastic leukemia undergoing the intensive phase or maintenance phase of chemotherapy vs. healthy controls	Unstimulated saliva collected by passive drooling after a rinse with distilled water	Fisher Scientific 10140 Indicator Paper	Level 4
Dubey et al. (2018) [8], Comparative study	Children (n = 90) five to 14 years olds undergoing treatment for acute lymphoblastic leukemia, type 1 diabetes mellitus, or asthma	pH strip immersed for 10 seconds in unstimulated saliva collected after one hour without eating or drinking	GC Saliva‐Check BUFFER kit	Level 4
Swapna et al. (2013) [9], Cross-sectional study	Patients (n = 97) with and without diabetes undergoing hemodialysis for more than a year	Unstimulated whole saliva pooled on the top of the tongue by the patient, and a pH paper strip was placed on it	Unspecified	Level 4
Díaz Rosas et al. (2018) [10], Descriptive and correlational study	Children (n = 60) with diabetes mellitus type 1 or type 2	Stimulated saliva collected after chewing a piece of parafilm and spitting repeatedly for five minutes	CIVEQ^®^	Level 4
Rai et al. (2011) [11], Case-control study	Six- to 12-year-old type I diabetic children (n = 200) vs. healthy children	pH tested on unstimulated saliva collected by passive drooling after two hours without eating or drinking	Indikrom Papers, GlaxoSmithKline Pharma, Mumbai, India, pH range between 4.5 and 6	Level 3
Schäfer et al. (2003) [12], Randomized parallel design study	Healthy adults (n = 21) in the prevention of oral health	By licking a sampling pad on the test trip and folding it on the pH indicator pad, for one group; no measurement of pH for the other group	pH range of 6.5-10	Level 2
Aliakbarpour et al. (2021) [13], Case-control study	Three- to 5-year-old children (n = 90) with ECC vs. children with severe ECC vs. caries-free children	Unstimulated whole saliva collected after at least an hour without drinking and eating	Merck, Germany	Level 3
Muchandi et al. (2015) [14], Case-control study	Children (n = 50) with severe ECC vs. caries three- to five-year-old children	Unstimulated whole saliva was collected by the draining method	Qualigens, Glaxo India Ltd., Mumbai, India	Level 3
Arendorf and Addy (1985) [15], Longitudinal study	Eight- to 17-year-old children (n = 33) with upper removable appliance orthodontic treatment	Saliva pH was measured by placing pH paper on the tongue for one minute	Whatman BDH pH indicator strips	Level 4
Walliczek-Dworschak et al. (2017) [16], Case-control study	Patients (n = 81) with taste disorders vs. healthy controls	Stimulated saliva spat out after chewing on a piece of paraffin for five minutes; pH measured on samples stored at -80 °C	Rebasit, Dr. Welte Pharma	Level 4
Caruso et al. (2016) [17], Case-control study	Patients (n = 20) with symptoms of GERD vs. healthy controls	Unstimulated whole saliva collected after a fast and a water rinse	Strip of pH 1-14 range Macherey-Nagel map	Level 3
Akbarnejad et al. (2022) [18], Case-control study	Three- to six-year-old children (n = 68) with β-thalassemia major vs. healthy controls	Unstimulated whole saliva collected after at least an hour without drinking and eating	Merck, Germany	Level 3

**Figure 2:**
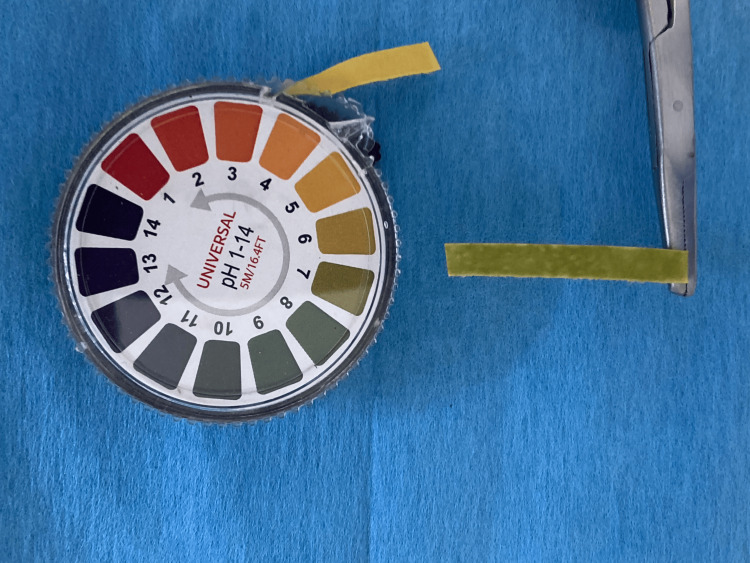
pH (6.5) measured with universal pH paper 1-14, 5M/16.4FT Image credit: Alp Alantar

**Figure 3:**
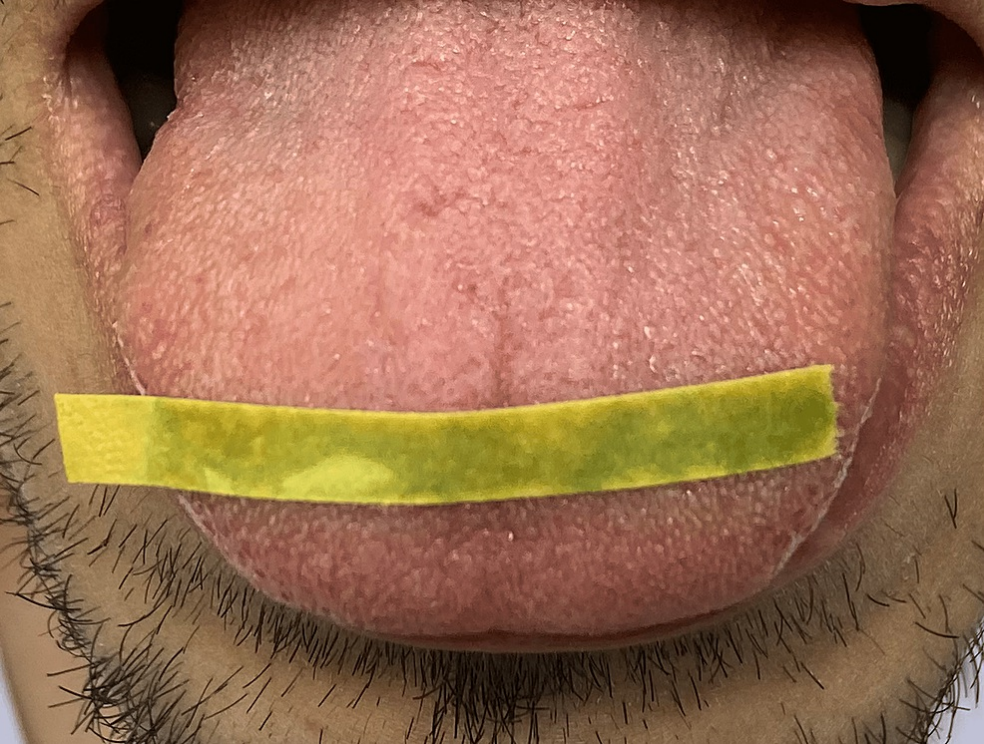
pH paper strip placed directly on the tongue Image credit: Baraa Shamsi-Basha

It appears that the more recent studies used a method of placing the pH paper in the collected saliva rather than placing the pH paper (Figure 2) directly in the mouth of the patient (Figure 3).

The majority of the studies measured unstimulated saliva which requires at least one hour of fasting. The brand of the pH contact varies widely from one study to another.

Indications of Salivary pH Indicator Paper Strip

In eight of the 15 articles, the studied populations were children. Five articles focused on diabetes. All studies on caries were carried out in children (Table 2).

**Table 2:** Relevance of pH to various pathologies ECC, early childhood caries; GERD, gastroesophageal reflux disease

Author	Pathology/treatment	Results	Significant results
Adults			
Laudenbach et al. (1974) [1]	Various pathologies	No statistical analysis	
Galgut (2001) [4]	Gingivitis and periodontitis	Mean pH in pockets: 7.02; mean pH in inflamed gingivae: 6.87; mean pH in healthy gingivae: 6.73; mean pH in the buccal floor: 6.61; mean pH in soft palate: 6.22	Yes
Ramya et al. (2015) [6]	Cancer	Patients before treatment: 46.70% pH 6-7 and 30% pH 4-6; patients undergoing treatment: 43.30% pH 6-7 and 23.30% pH 4-6; healthy controls: 66.70% pH 7-8 and 16.70% pH 6-7	No
Swapna et al. (2013) [9]	Hemodialysis patients with or without diabetes	Patients with diabetes: mean pH 7.14; patients without diabetes: mean pH 7.02	No
Schäfer et al. (2003) [12]	Prevention of oral health	An increase in motivation relating to oral hygiene was found in the test group who had been using the saliva test strip	Yes
Walliczek-Dworschak et al. (2017) [16]	Taste disorders	Patients with taste disorders: mean pH 6.9; healthy controls: mean pH 6.9	No
Caruso et al. (2016) [17]	GERD	Patients with GERD: average pH 4.9; patients without GERD: average pH 6.7	Yes
Children			
Kapoor et al. (2019) [7]	Leukemia	Leukemia patients: mean pH 6.17; patients under intensive treatment: mean pH 5.86; patients under maintenance treatment: mean pH 6.48; healthy controls: mean pH 7.46	Yes
Dubey et al. (2018) [8]	Leukemia, type I diabetes, and asthma	Chemotherapy: mean pH: 5.0-5.8; insulin therapy: mean pH: 6.0-6.6; β2-agonists inhalers: mean pH 6.8-7.8	Yes
Díaz Rosas et al. (2018) [10]	Diabetes	Mean pH: 6.76	Yes
Rai et al. (2011) [11]	Type I diabetes	Diabetic children: mean pH: 6.406; control group: mean pH 7.071	Yes
Aliakbarpour et al. (2021) [13]	ECC	ECC group: mean pH 7.03; severe ECC group: mean pH 6.95; caries-free group: mean pH 7.27	No
Muchandi et al. (2015) [14]	Severe ECC	Severe ECC group: mean pH 6.42; caries-free group: mean pH 7.46	Yes
Arendorf and Addy (1985) [15]	Orthodontic appliance therapy	Mean pH before treatment: 7.36; mean pH during treatment: 6.89; mean pH after treatment: 7.61	Yes
Akbarnejad et al. (2022) [18]	β-thalassemia major	Case: mean pH 6.57; control: mean pH 7.25	Yes

Head and Neck Cancer and Hematological Pathology

Ramya et al. [6] measured a lower pH for patients with cancer before or under treatment compared to healthy controls. A significant decrease in salivary pH for patients with cancer was also found, but this resulted from data measured by a pH meter; the results of the pH paper were not statistically analyzed. Moreover, it was not specified which cancer was involved, although we can assume they were head and neck cancers.

Cervicofacial radiotherapy is also reported to change the pH to be more acidic, as Laudenbach et al. [1] pointed out, but this publication did not perform statistical analysis.

Kapoor et al. [7] studied the pH of children with leukemia and found a significantly lower pH in the leukemia group as well as in the intensive treatment group compared to the maintenance treatment group. The children with leukemia had fewer caries compared with the controls. Dubey et al. [8] examined the salivary pH of patients with leukemia; the authors compared caries status using mean decayed, missing, and filled teeth (DMFT) indices with salivary pH. Results revealed that the mean deft/DFMT indices of children with leukemia were significantly higher than children with diabetes or asthma, meaning children with leukemia had poorer oral health. However, this study did not use healthy patients as a control and did not mention the salivary pH measured. In the oncological population, it appears that good oral hygiene is important in order to maintain the salivary pH in a healthy 7.0 mean range [6].

Diabetes

In Swapna et al.’s study [9], there was no statistically significant difference in salivary pH between the two groups of the patients’ diabetics and nondiabetics under hemodialysis; there was a suggestive significance statistically, with a pH value of >7.0 being recorded among 34% nondiabetics and only 17% diabetic patients (p < 0.056). No correlation was analyzed with other oral manifestations or oral health.

A low pH in the case of diabetes was also described by Laudenbach et al. [1], with causes such as hyposialia or a tendency to ketonemia.

In the pediatric population, pH was measured at around 6.76 in diabetic patients by Díaz Rosas et al. [10], and they obtained a positive correlation between salivary flow and pH. Rai et al. [11] measured a significantly lower salivary pH in diabetic children compared to healthy children, as well as poorer oral hygiene, but no correlation was found between pH and oral health.

Periodontitis and Gingivitis

Diagnosis of periodontitis needs recording clinical factors: gingival health, pocket depths, bleeding on probing, furcation lesions, tooth mobility, and occlusion. Having recorded these factors, together with information on the patient’s age and medical history, a provisional diagnosis can be made. Additional information may be obtained from a radiographic examination, a pulp vitality test, and microbiological and hematological investigations. Galgut [4] established a highly significant correlation between total pocket and pH range and a significant correlation between deep pocket and low pH range. No significant correlation between moderate pocket and pH, or between healthy sites, gingivitis, and pH. In this study, the pH varied widely from one measure to another, and the authors concluded that a more precise way of measuring pH was needed, but that the pH paper can be a useful technique for screening the disease activity in clinical practices. The knowledge of salivary pH levels helps justify the prescription of sodium bicarbonate-containing solutions or dentifrices.

Laudenbach et al. [1] also reported an alkaline pH in the case of parodontitis, stomatitis, or other oral bacterial infections such as parotitis or sialodochitis.

Oral Health

The use of pH paper by the patients themselves in their hygiene routine showed a significant increase in their motivation to brush their teeth better and longer [12]. pH paper can be a tool to increase the motivation of patients to take care of their oral health.

About caries in children, Aliakbarpour et al. [13] concluded that there was no significant difference in mean salivary pH between children with or without ECC. They mentioned the lack of sensitivity of pH paper and the possible error in reading the results as possible causes for this outcome. Muchandi et al. [14] found different results, with a pH significantly more acidic in children with severe ECC compared to caries-free children and a negative correlation between salivary pH and the total antioxidant capacity.

Orthodontic Treatment

Arendorf and Addy [15] found a significant fall in salivary pH during orthodontic appliance therapy and a significant rise after the appliance was removed. It was associated with a significant increase in the prevalence of candida carriage during the appliance treatment and a significant decrease after removal.

Taste Disorders

In their study, Walliczek-Dworschak et al. [16] did not find a significant difference in salivary pH between the patients and the controls. A significant correlation was established between pH and salivary flow rate, and patients showed a higher flow rate.

Gastroesophageal Reflux Disease (GERD)

Caruso et al. [17] found a significant decrease in salivary pH for patients with GERD. They concluded that a presumptive diagnosis of GERD can be made with a salivary pH ≤5 associated with a nasal pH ≥8. This implies that the method of measurement of the nasal pH must be known by the practitioner.

Β-Thalassemia Major

Akbarnejad et al. [18] found a pH significantly lower in children with β-thalassemia than in healthy controls. The study did not establish a correlation between pH acidity and dental health.

Discussion

Reliability of Salivary pH Measurement Using pH Paper

The majority of the studies measured unstimulated saliva, which requires at least one hour of fasting. This is a limit for this method of pH measurement in consultations because it withdraws from the fast and practical side of the exam. The reliability of pH paper compared to a pH meter has also been examined for other biological fluids such as vaginal secretions [19] or aspirated gastric juice [20], and it was shown that pH determination by pH paper was reliable. Song et al. evaluated the reliability of salivary pH measurement using pH paper. They concluded that pH paper has sufficient reproducibility, especially for unstimulated saliva [21].

This suggests that pH paper can be used as a support for clinical examination, followed by a more precise evaluation if necessary. The pH paper test is a low-cost, noninvasive method that ensures an instantaneous result that is immediately visualized by the patient. Thus, the training of medical professionals will allow for accurately performing pH paper testing and interpreting results in the context of specific patient conditions.

Indications of contact salivary pH paper

Oral Medicine 

The use of salivary pH in oral medicine as a tool in diagnostic and therapeutic assessment could be summarized as follows:

ECC: pH paper may be used as a quick and noninvasive chairside test to assess salivary pH levels in young children (before five years old), particularly those at risk of ECC. Performing regular salivary pH testing during dental checkups allows for the identification of acidic conditions conducive to ECC development.

Oral lesions: Salivary buffering capacity depends on the amount of acid and bases present in the secreted saliva, and bicarbonate is the principal buffering agent. Bicarbonate secretion increases as the salivary flow rate increases. Foglio-Bonda et al. [22] found statistically significant decreased salivary flow rate and altered pH in patients with oral lesions when compared to patients without oral lesions. In their study, a decreased flow rate of 0.336 ml.min-1 and an increased pH of 6.69 were seen.

Gingivitis and periodontitis: Using pH papers as a rapid diagnostic tool to assess salivary pH in patients with uncontrolled severe (grades 3 and 4) periodontitis and implementing salivary pH testing as a supplementary measure to evaluate the oral environment and its impact on periodontal health status can be beneficial. Salivary pH analysis facilitates the discrimination between inflamed and healthy gingival tissues and provides a means to evaluate pH variations within periodontal pockets. Early diagnosis of salivary low pH range in children as well as periodontitis with deep pocket associated with a low pH range in adults should lead to the supply of an amount of fluoride and the prescription of sodium bicarbonate-containing dentifrices, respectively [23]. In children, the use of a chewable toothbrush may help reduce plaque and elevate salivary pH [24].

Taste disorders: Salivary pH testing demonstrates limited discriminative ability between individuals with taste disorders and unaffected controls.

General Medicine

The use of salivary pH in general medicine as a tool in diagnostic and therapeutic assessment could be summarized as follows:

Head and neck cancer patients: Salivary pH monitoring with pH strips presents potential utility in tracking radiotherapy-induced pH fluctuations among cancer patients both pre- and intra-treatment [6].

GERD patients: The utilization of salivary pH paper aids in discerning between GERD-afflicted individuals and controls by evaluating their mean salivary pH levels. The saliva pH, which under normal conditions varies between 7.0 and 7.2 in the presence of GERD, is affected by the influence of acidic gastric juice [17].

Diabetic patients undergoing hemodialysis and diabetic children: Assessment of salivary pH dynamics offers valuable insights into their oral health status [6,8].

β-thalassemia major: For patients with β-thalassemia major, salivary pH examination enables differentiation between β-thalassemia major cases and controls based on mean salivary pH levels; pH is significantly lower compared to the control group [18].

Leukemia: Salivary pH paper distinguishes between leukemia patients undergoing varied treatment modalities and their healthy counterparts [7]. Results revealed lower pH mean values in children diagnosed with leukemia undergoing chemotherapy compared with healthy children without systemic disease. Moreover, a lower pH mean value was noticed in children with leukemia undergoing the intensive phase of chemotherapy compared with children with leukemia undergoing the maintenance phase of chemotherapy.

## Conclusions

The lack of sensitivity of pH paper must be underlined. A low pH is a cofactor leading to oral pathologies, and the use of pH paper constitutes an easy diagnostic instrument in patients with pH variations correlated to leukemia, diabetic mellitus, or orofacial radiotherapy. The evaluation of salivary pH using pH paper may be used as a quick chairside test, specifically in cases of ECC and uncontrolled severe periodontitis. The knowledge of salivary pH level helps justify the prescription of fluoride, sodium bicarbonate-containing solution, or dentifrices, respectively.

## Appendices

Appendix A: Members of the working group and lecture committee

The members of the working group are as follows: Baara Shamsi-Basha (Oral Surgery Unit, F-92000, Nanterre), Raphaëlle Bernard-Garbati (Oral Surgery Unit, F-92000, Nanterre), Emmanuel Mortier (Internal Medicine Unit, F-92000, Nanterre), Anne Grasland (Rheumatology Unit, F-92000, Nanterre), Karim Lachgar (Diabetology Unit, tF-92000, Nanterre), and Alp Alantar (Oral Surgery Unit, F-92000, Nanterre).

The members of the lecture committee are as follows: Fateh Al Chalabi (Orthodontics, F-9200 Nanterre), Anne-Françoise Batto (Biology, Data Scientist, INSERM U1138), Jacques-Christian Béatrix (Oral Medecine, 77796, Nemours), Delphine Beaudouvie (Nursery, F-92000, Nanterre), I. Benlekhal (Oral Surgery Unit, F-92000, Nanterre), Alain Bery (Orthodontist-France), Jean-Batiste Charrier (Stomatology and Maxillofacial Surgery, F-75006, Paris), Alex Clement (Oral Surgery, F-92000, Nanterre), Chatel C. (Oral Medicine, F-38000, Grenoble), Isabelle Incigül Conrad (Radiology, F-78105, Saint Germain en Laye), Martine Dame (Oral Medicine, Oral Surgery Unit, F-92000, Nanterre ), Michel Guyot (Oral Surgery, F-85200, Fontenay-le-Comte), Sylvie Jublot (Prosthesis, F-92000, Nanterre), Arnaud Lafon (Oral Surgery, F-69001, Lyon), Louis Maman (Oral Surgery, F-94200, Ivry/s/Seine), Guy Marti (Stomatology and Maxillofacial Surgery, F-77000, Melun), Bérengère Phulpin (Oral Medicine, F-54035, Nancy), Sylvie Rajca (Gastroentrology and Hepathology, F-92000, Nanterre), Eliane Snaoui (Nursery, F-92000, Nanterre), Marc Sorel (Internal Medicine, F-77796, Nemours), Guy Princ (Stomatology and Maxillofacial Surgery, F-75116, Paris), Sylvie Rajca (Gastroenterology and Hepathology), Marion Renoux (Oral Surgery, 61100, Flers), Mohamed Y. Kharma Sr. (Maxillofacial Surgery), and Yvan Smatt (Stomatology and Maxillofacial Surgery, F-75016, Paris).
